# Fabrication of Highly Conductive Inkjet Printing Silver Nanoparticle Ink via a Synergistic Strategy Combining Centrifugal Classification and Dispersant Optimization

**DOI:** 10.3390/ma19030628

**Published:** 2026-02-06

**Authors:** Guo-Xiang Zhou, Yan Wang, Xing-Ping Zhou, Kuang Zhang, Zhi-Hua Yang, De-Chang Jia, Yu Zhou

**Affiliations:** 1 Department of Microwave Engineering, Harbin Institute of Technology, Harbin 150001, China; zhangkuang@hit.edu.cn; 2 Chongqing Research Institute of HIT, Chongqing 401135, China; wy010721@163.com (Y.W.); zhouxingping@enchen-tech.com (X.-P.Z.); dcjia@hit.edu.cn (D.-C.J.); 3 Institute for Advanced Ceramics, School of Materials Science and Engineering, Harbin Institute of Technology, Harbin 150080, China; zhouyu@hit.edu.cn; 4 School of Materials Science and Engineering, Harbin Institute of Technology Shenzhen, Shenzhen 518055, China

**Keywords:** silver nanoparticle inks, inkjet printing, centrifugal classification, dispersion method, conductivity, printed electronics

## Abstract

**Highlights:**

**What are the main findings?**

- A synergistic strategy integrating centrifugal classification, high-pressure homogenization, and dispersant optimization was successfully developed.
- The resulting optimized Ag NP ink demonstrated excellent jetting stability (clogging-free) and suitable rheology for reliable inkjet printing.
- A high electrical conductivity of 1.506 × 10^7^ S/m was achieved after low-temperature sintering at only 260 °C.
- Using Pluronic F127 as the sole dispersant allowed its complete removal during sintering, promoting the formation of a dense conductive network.

**What are the implications of the main findings?**

- The strategy provides a scalable and practical manufacturing pathway for producing high-performance printed electronics.
- The low-temperature sintering capability (260 °C) makes the ink compatible with heat-sensitive flexible substrates.
- The complete removal of the dispersant eliminates insulating residues, which is crucial for achieving high conductivity in printed metal features.

**Abstract:**

Inkjet printing technology shows significant potential for producing high-performance conductive circuits in printed electronics. However, conventional silver nanoparticle (Ag NP) inks often face challenges such as nozzle clogging, poor stability, and low conductivity after low-temperature sintering. While most existing studies focus solely on dispersant selection or individual process optimization, few have systematically explored the synergistic effects of particle size distribution, dispersion methods, and dispersant dosage. This study proposes a sequential optimization approach involving centrifugal classification to identify an optimal Ag NPs source and size distribution, followed by comparison and optimization of different dispersion methods. Furthermore, the effects of dispersant (a PEO-PPO-PEO triblock copolymer) concentration and application strategy (individual or combined use) on the rheological properties and conductivity of the ink were systematically investigated. The optimized Ag NP ink demonstrated excellent jetting stability with no nozzle clogging, exhibiting a surface tension of 19.60 mN/m and a viscosity of 6.83 mPa·s. After low-temperature sintering at 260 °C on glass or polyimide (PI) substrates, the printed patterns achieved a high electrical conductivity of 1.506 × 10^7^ S/m. Printing on polyethylene terephthalate (PET) at 150 °C confirmed compatibility with heat-sensitive flexible substrates. This work offers a comprehensive and practical strategy for developing highly reliable and conductive Ag NP inks, facilitating their application in next-generation printed electronics.

## 1. Introduction

The proliferation of flexible and wearable electronic devices has intensified the demand for compatible, low-cost manufacturing technologies [1,2,3]. Among various techniques, inkjet printing stands out as a promising digital, non-contact, and additive method for patterning functional materials onto flexible substrates, offering advantages such as high material utilization, design flexibility, and compatibility with roll-to-roll processes [4,5,6]. The performance of printed electronic devices is predominantly determined by the properties of the conductive inks [7,8].

Silver-based inks, particularly those containing silver nanoparticles (Ag NPs), are widely investigated due to silver’s excellent electrical conductivity, chemical stability, and oxidation resistance [9,10]. Despite these advantages, the practical applications of Ag NP inks face significant challenges. The high surface energy of Ag NPs promotes agglomeration, leading to poor colloidal stability over time, sedimentation, and ultimately nozzle clogging during the printing process, which compromises print reliability and printhead lifespan [11,12]. Furthermore, the thermal sensitivity of common polymer substrates (e.g., PET, PI) necessitates low-temperature sintering (<300 °C) [13,14]. At these temperatures, incomplete removal of organic dispersants/stabilizers and insufficient particle coalescence often result in highly porous microstructures with elevated electrical resistance, which is substantially inferior to the conductivity of bulk silver or films sintered at high temperatures [15,16,17].

Conductive silver inks are generally categorized into two types: silver nanoparticle (SNP) inks [18] and silver organic complex (SOC) inks [19], each with distinct trade-offs. SNP inks, stabilized by surfactants or polymers (e.g., PVP, PAA) via steric or electrostatic mechanisms, offer good chemical stability but often suffer from high viscosity, aggregation, and rough sintered surfaces [20]. SOC inks, being particle-free, allow for smooth films and high-resolution patterning but are susceptible to environmental factors and prone to cracking during decomposition [21].

Research efforts have explored various strategies to address these issues, including optimizing dispersants [22,23], developing composite structures [24,25], and employing multi-scale particles [26,27]. However, many studies focus on optimizing a single parameter, overlooking the critical synergies between initial particle size distribution, efficient dispersion methodologies and dispersant selection/application strategy. This lack of a holistic approach hinders the simultaneous achievement of long-term stability, reliable inkjet printing behavior, and high electrical conductivity after low-temperature sintering.

In this work, Pluronic F127 (a PEO-PPO-PEO triblock copolymer) is used as the primary dispersant due to its excellent steric stabilization capability in mixed aqueous/organic solvents and, more importantly, its relatively low thermal decomposition temperature (~322 °C). This allows for near-complete removal during low-temperature sintering, unlike common stabilizers such as polyvinylpyrrolidone (PVP), which persist at temperatures below 300 °C and leave insulating residues that impede conductivity [28,29].

To address this gap, it is essential to investigate the interrelationships and mechanisms underlying three critical aspects. This study aims to develop high-performance Ag NP ink by adopting a systematic and collaborative strategy. The effects of Ag NPs size classification, dispersion methods, and Pluronic F127 application strategy on the ink’s stability, conductivity, viscosity, and surface tension were explored. Subsequent optimization of process and formulation parameters yielded an Ag NP ink with superior printability and high electrical conductivity, suitable for printed circuits. The outcomes offer theoretical insight and experimental support to facilitate the industrial production and broader application of Ag NP inks.

## 2. Materials and Methods

### 2.1. Materials

Silver nanoparticles (Ag NPs, ~50 nm) were purchased from Beijing BroadTeko Intelligent Technology Co., Ltd. (Beijing, China), Brofos Nanotechnology (Ningbo) Co., Ltd. (Ningbo, China), and Shanghai Pantian Powder Material Co., Ltd. (Shanghai, China). Pluronic F127 was supplied by Sigma-Aldrich (Shanghai) Trading Co., Ltd. (Shanghai, China) Dioctyl sulfosuccinate sodium salt (AOT), triethylene glycol monomethyl ether (TEGME), polyvinylpyrrolidone (PVP, K30), glycerol and polyvinyl butyral (PVB) were obtained from Shanghai Aladdin Biochemical Technology Co., Ltd. (Shanghai, China). Defoamer K155 was provided by Dongguan Heli Chemical Trade Co., Ltd. (Dongguan, China) Anhydrous ethanol, isopropanol (IPA) and 2-(2-ethoxyethoxy)ethyl acrylate were purchased from Chongqing Chuandong Chemical (Group) Co., Ltd. (Chongqing, China) Deionized (DI) water was used in all the experimental processes.

### 2.2. Ag NP Ink Formulation and Optimization Strategy

**Figure 1:**
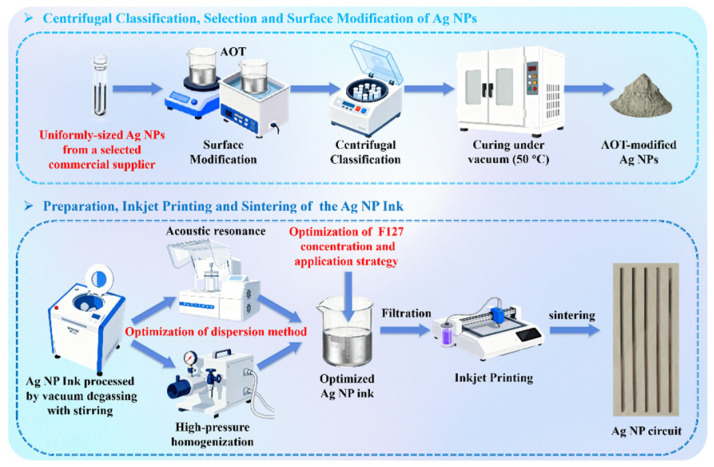
Schematic illustration for the preparation process of optimized Ag NP ink.

The fabrication of the highly conductive Ag NP ink involved a sequential optimization strategy, as illustrated in Figure 1, comprising: (1) centrifugal classification for selecting optimal Ag NPs from commercial suppliers, (2) surface modification, (3) dispersion method optimization and (4) dispersant (F127) concentration and application strategy optimization.

#### 2.2.1. Centrifugal Classification and Surface Modification

Commercial Ag NPs from different suppliers were initially dispersed in DI water (10 wt.%) via magnetic stirring for 30 min. The suspensions were centrifuged (1000~3000 r/min, 20 min) to isolate particles with a narrow size distribution. The optimal Ag NPs source and centrifugal speed were selected based on sedimentation stability and conductivity (after drying at 150 °C), as detailed in Section 3.1. The selected Ag NPs were subsequently surface-modified with AOT (1 wt.% relative to Ag NPs) to enhance compatibility. The mixture was stirred for 24 h at room temperature, ultrasonicated (30 min, <40 °C), and then recovered by centrifugation (5000 r/min, 20 min), followed by vacuum drying at 50 °C for 90 min.

#### 2.2.2. Ink Preparation and Dispersion Optimization

The detailed compositions of the key ink variants studied in this work—the PVP-containing base ink, the optimized F127-only ink, and an F127/PVP composite ink—are summarized in Table 1. A general preparation procedure was followed for all variants with specific adjustments noted below.

First, surface-modified Ag NPs (fixed at 30 wt.%) were dispersed. For the PVP-containing formulations (base and composite inks), this was done by incorporating the Ag NPs into a PVP/TEGME solution (PVP concentration as specified in Table 1). For the F127-only optimized ink, the Ag NPs were directly blended with a pre-prepared solution of F127 in a water/IPA/ethanol mixture (e.g., F127 concentration: 10 wt.% of total ink).

Subsequently, common additives—glycerol (2.5 wt.%), 2-(2-ethoxyethoxy)ethyl acrylate (0.1 wt.%), PVB (0.1 wt.%), and defoamer K155 (0.2 wt.%)—were added to all formulations. For the composite ink, the required amount of F127 (5 wt.%) was added at this stage via its stock solution. Each mixture was then stirred at 800 r/min for 3 h and vacuum-degassed to obtain the primary ink suspension.

For the dispersion method optimization study detailed in Section 3.2, the PVP-containing base ink (as defined in Table 1) was used as the starting formulation. This ink was further processed using either a high-pressure homogenizer (HPH, 2000 bar, 30 min) or an acoustic resonance mixer (AR, at an acceleration of 90 g, 30 min). The resulting suspension was filtered through a 0.45 μm membrane to yield the final ink for evaluation.

#### 2.2.3. Dispersant Concentration Optimization

To investigate the effect of dispersant concentration (Section 3.3), the F127 content was varied (6, 8, 10, 12, and 14 wt.%) while keeping other components constant. The influence of the dispersant system (F127 alone versus F127/PVP composite) on the conductivity after sintering at different temperatures (200, 220, 240, and 260 °C for 1 h) was also studied (Section 3.4).

### 2.3. Inkjet Printing Process

The Printability was evaluated using a customized piezoelectric inkjet printing system equipped with a 30 μm diameter nozzle. The ink cartridge was maintained at 25 °C, and glass, PI, and PET substrates were heated to 25 °C. A bipolar driving waveform with 21 V amplitude and 30 μs pulse width was applied at a firing frequency of 30 kHz. Before printing, the ink was filtered through a 0.45 μm syringe filter and vacuum-degassed for 10 min. To assess jetting stability and nozzle clogging, continuous printing was performed for 2 h. The printed silver nanoparticulate films on glass and PI substrates were subsequently sintered at 260 °C to achieve high electrical conductivity. In contrast, the films on PET substrates were sintered at a lower temperature of 150 °C to prevent thermal deformation of the flexible substrate, primarily to demonstrate the printing feasibility on flexible surfaces.

### 2.4. Characterization

The ink density was measured using a pycnometer at 25 °C. The morphology of Ag NPs and dispersant coating was observed by Transmission Electron Microscopy (TEM, FEI Tecnai F20, Hillsboro, OR, USA). The surface morphology of the conductive ink suspensions and the sintered films was examined by Scanning Electron Microscopy (SEM, TESCAN MIRA3 LMH, Brno, Czech Republic), which was also employed for the measurement of the film thickness after sintering. Particle size distribution was analyzed with a Laser Particle Size Analyzer (Bettersize 2600, Dandong, China). Electrical conductivity was determined by first measuring sheet resistance (R_s_) directly using a Four-Point Probe Resistivity Measurement System (HPS 2661, Changzhou Helpass Electronic Technologies Inc., Changzhou, China). Resistivity (ρ) was calculated as ρ = R_s_ × t, and conductivity as σ = 1/ρ. All reported conductivity values are the mean of five independent measurements on different printed samples. Surface tension was characterized using a Contact Angle/Surface Tensiometer (Dataphysics OCA20, Filderstadt, Germany). The thermal decomposition behavior of the dispersant systems (F127 alone versus an F127/PVP composite) was characterized by simultaneous thermogravimetric analysis and differential scanning calorimetry (TGA-DSC; HITACHI STA300, Tokyo, Japan). Fourier-transform infrared spectroscopy (FTIR, Nicolet iS50, Thermo Fisher, Waltham, MA, USA) was used to analyze the presence of residual organics on printed films before and after sintering.

The Rheological properties of the inks were characterized using a rotational rheometer (HAAKE MARS40, Thermo Scientific, Karlsruhe, Germany) equipped with a concentric cylinder geometry and a temperature-controlled Peltier system. All measurements were performed at 25.0 ± 0.2 °C. A constant gap of 4 mm was maintained. To ensure sample uniformity and erase loading history, each measurement began with a pre-shear step at 100 s^−1^ for 2 min, followed by a 1 min equilibration period. Steady-shear viscosity was measured over a shear rate range of 30 to 60 s^−1^. This range was selected as it is representative of the bulk flow conditions in the ink supply lines immediately before droplet ejection—a critical stage for ensuring reliable jetting—as discussed in the context of inkjet printing processes [30]. The specific value in the text (6.83 mPa·s) is the mean of triplicate measurements at 40 s^−1^. Oscillatory measurements were performed within the linear viscoelastic regime, confirmed by prior amplitude sweeps (0.1–100% strain at 1 Hz). A fixed strain amplitude of 1% and frequency of 1 Hz were used for all frequency and temperature sweep measurements. Temperature sweeps to determine gel-sol transitions were conducted at a heating rate of 1.5 °C/min. All rheological data reported are the mean of triplicate measurements performed on freshly loaded samples. The temporal stability of the ink’s rheology was assessed by monitoring its viscosity after 7 days of storage at 25 °C.

## 3. Results and Discussion

### 3.1. Optimization of Ag NPs Selection via Centrifugal Classification

**Figure 2:**
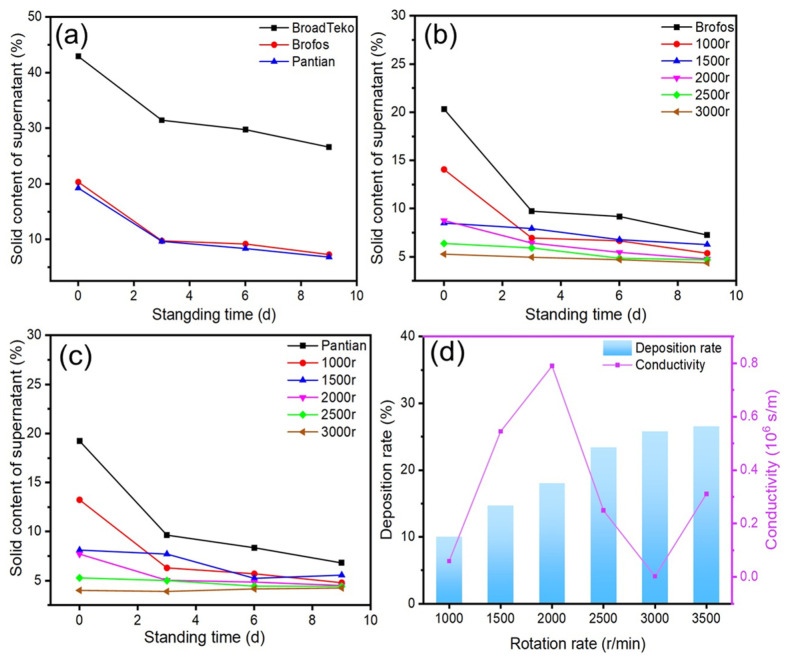
Effects of centrifugal processing on the sedimentation behavior, electrical conductivity, and particle size distribution of Ag NPs: (**a**) Sedimentation curves of Ag NPs from different suppliers after ultrasonic treatment, presented as the silver content in the supernatant versus time; (**b**,**c**) Sedimentation curves of Brofos and Pantian Ag NPs under different centrifugation speeds, showing the silver content remaining in the supernatant over time; (**d**) Deposition rate and conductivity of Brofos Ag NPs as functions of centrifugation speed.

The initial selection of Ag NPs with a narrow size distribution is crucial for ink stability. The dispersion stability of Ag NP suspensions from three commercial Ag NPs sources (BroadTeko, Brofos, and Pantian) was evaluated by monitoring the silver content in the supernatant during static storage (Figure 2a). After 9 days, the sedimentation rates for Brofos and Pantian Ag NP suspensions were similar (~35%), significantly lower than that for BroadTeko Ag NP suspensions (62%), indicating superior intrinsic dispersion stability. This disparity is attributed to the broader particle size distribution of BroadTeko Ag NPs, which promotes agglomeration and sedimentation [31,32].

Further centrifugal processing (1000~3000 r/min) was applied to Brofos and Pantian Ag NP suspensions. Figure 2b,c show that higher centrifugal speeds improved dispersion, reducing sedimentation rates over time, with the effect being more pronounced at higher speeds. After 8 days, sedimentation equilibrium was approached. The Brofos Ag NP suspension exhibited a clear plateau in its sedimentation curve within the 2500~3000 r/min range, indicating excellent anti-settling properties, whereas the Pantian Ag NP suspension’s curve declined slowly, likely due to weaker surface modifier-solvent interactions.

Based on these results, Brofos Ag NPs were selected for further study. The effect of centrifugal speed (1000~3000 r/min) on deposition rate and conductivity of the Brofos Ag NP suspension is shown in Figure 2d. The deposition rate increased with speed, but conductivity exhibited a non-monotonic relationship. A conductivity maximum (0.8 × 10^6^ S/m) was observed at 2000 r/min. Higher speeds (e.g., 3000 r/min) removed excessive finer or well-dispersed nanoparticles, drastically reducing conductivity (0.2 × 10^4^ S/m). This suggests that moderate centrifugation aids dispersion and conductive network formation, while excessive speed causes detrimental over-agglomeration [33,34].

**Figure 3:**
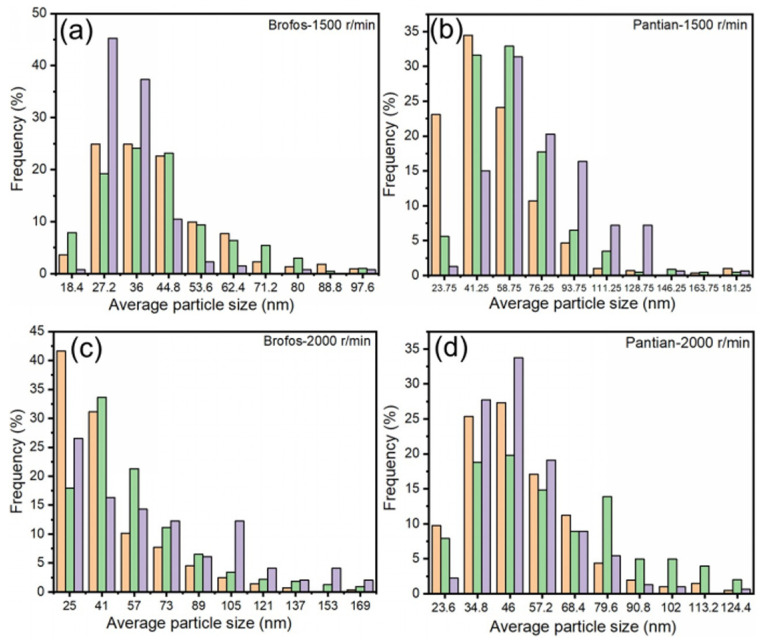
Size distribution of Ag NPs in the supernatant of Brofos and Pantian Ag NPs after centrifugation: (**a**,**b**) at 1500 r/min; (**c**,**d**) at 2000 r/min.

Particle size analysis of the supernatants after centrifugation at 1500 and 2000 r/min (Figure 3a–d) revealed that most particles were below 100 nm. Quantitative analysis showed that over 70% of Brofos Ag NPs were below 50 nm at both speeds, compared to less than 50% for Pantian, confirming the narrower size distribution and superior homogeneity of Brofos Ag NPs. The fraction of sub-50 nm Brofos Ag NPs increased further at 2000 r/min, indicating enhanced separation and enrichment of smaller particles at higher centrifugal forces.

Balancing stability and conductivity, 1500 r/min (18% deposition rate) was chosen as the optimal centrifugal condition. Under this condition, Brofos Ag NPs exhibited a narrow size distribution (>70% <50 nm), effectively minimizing sedimentation stratification caused by size disparity and providing a foundation for highly stable ink.

### 3.2. Comparison of the Dispersion Methods

Following the selection of optimally sized Ag NPs, the efficacy of different dispersion methods in achieving a stable and printable ink was evaluated using the PVP-containing base ink formulation (Table 1) as the precursor. Two dispersion methods were compared: high-pressure homogenization (HPH) and acoustic resonance (AR).

HPH utilizes a high-pressure pump to force the suspension through a narrow valve, generating intense shear, cavitation, and impact forces that effectively disrupt agglomerates [35]. AR employs high-frequency mechanical vibrations to disperse particles primarily through cavitation effects, potentially enhanced by resonance at specific frequencies [36].

**Figure 4:**
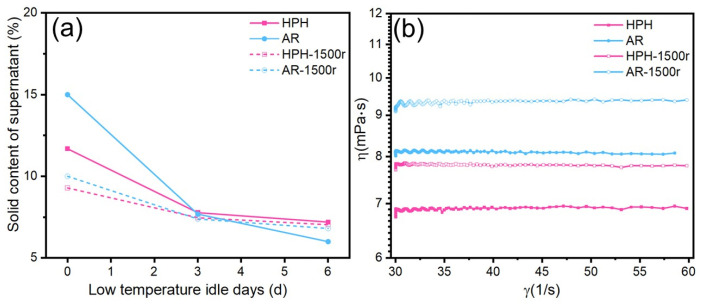
Comparison of Ag NP inks treated by different dispersion techniques: (**a**) Ag NPs content in the supernatant; (**b**) Viscosity of HPH-treated and AR-treated inks before and after centrifugation.

To evaluate dispersion efficacy, inks treated by HPH and AR (30 min each) were centrifuged (1500 r/min, 20 min) to remove unstable aggregates. HPH demonstrated superior performance in enhancing ink stability. Figure 4a shows that the solid content in the supernatant of the HPH-treated sample decreased to 61.5% of its initial value over 6 days of static storage, compared to a 60.0% decrease for the AR-treated sample. After centrifugation, the solid content loss was 24.3% for HPH versus 32.0% for AR, confirming HPH’s greater effectiveness in inhibiting re-agglomeration and ensuring long-term stability.

While the initial differences in supernatant solid content between HPH and AR treatments appear modest in Figure 4a, the key distinction lies in the ink’s resistance to re-agglomeration under stress. The HPH-treated ink exhibited significantly less solid loss after centrifugation (24.3% vs. 32.0% for AR) and maintained a higher solid content during prolonged static storage. This indicates that the intense shear forces generated by HPH not only break up initial agglomerates but also create a more electrosterically stable dispersion that resists re-formation of aggregates.

Rheological measurements provided further support for the advantage of HPH (Figure 4b). The HPH-treated ink had a lower initial viscosity (6.8 mPa·s) than the AR-treated ink (8.1 mPa·s). After centrifugation, the viscosity of both increased, but the HPH sample maintained a lower viscosity. The lower initial viscosity and superior viscosity stability afforded by HPH are crucial for maintaining reliable inkjet printing. The superior performance of HPH is attributed to the more intense mechanical forces generated, which more effectively break down strong agglomerates and create a more uniform and stable dispersion [37]. As shown in Figure 4b, the viscosity of the HPH-treated ink remained nearly constant across the measured shear rate range of 30–60 s^−1^, indicating near-Newtonian behavior that is favorable for consistent inkjet droplet formation.

### 3.3. Effect of F127 Concentration on Dispersion and Rheology

With the optimal Ag NPs source (Brofos) and dispersion method (HPH) established, the influence of the dispersant Pluronic F127 concentration was systematically investigated within the F127-only ink formulation framework. To maintain a total mass of 100 wt.%, the F127 concentration was varied (6, 8, 10, 12, and 14 wt.%) while keeping the content of all other components—specifically Ag NPs (30 wt.%), glycerol (2.5 wt.%), 2-(2-ethoxyethoxy)ethyl acrylate (0.1 wt.%), PVB (0.1 wt.%), and defoamer K155 (0.2 wt.%)—constant. The balance was adjusted by proportionally varying the primary solvent mixture of TEGME, DI water, IPA, and ethanol, following the ratio established in the optimized formulation (Table 1). This approach allowed for isolating the effect of F127 concentration on ink properties.

**Figure 5:**
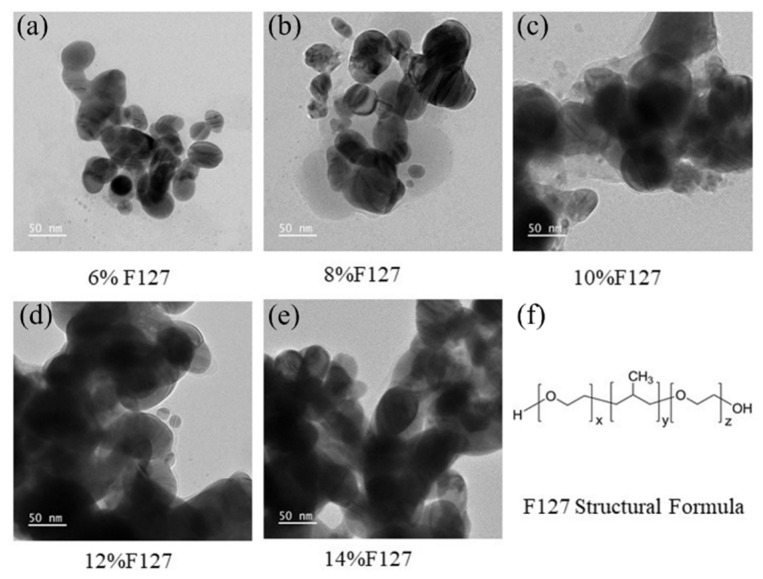
TEM images of silver nanoparticles coated with different concentrations of F127 dispersant and the molecular structure of F127: (**a**–**e**) Morphology and dispersion state of Ag NPs at F127 concentrations of 6%, 8%, 10%, 12%, and 14%, respectively; (**f**) Structural formula of Pluronic F127.

**Figure 6:**
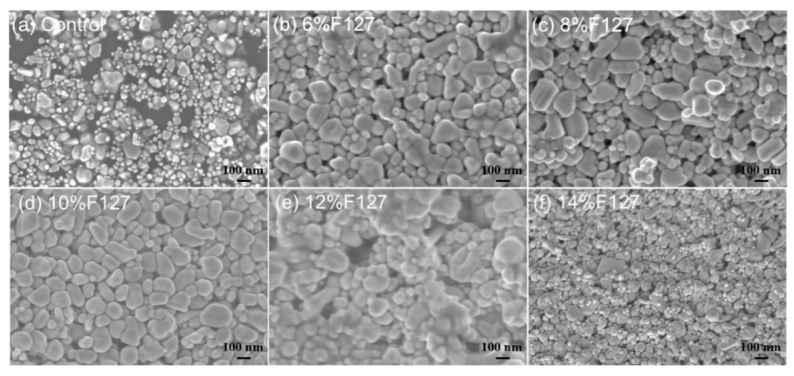
SEM images of particle morphology after centrifugal deposition of Ag NP ink with different F127 additions: (**a**–**f**).

The concentration of the dispersant Pluronic F127, a PEO-PPO-PEO triblock copolymer, is critical for achieving a balance between colloidal stability and post-sintering conductivity. Its hydrophobic PPO blocks adsorb onto the Ag NP surface, while the hydrophilic PEO chains extend into the solvent, providing steric stabilization [38]. TEM imaging confirmed the successful adsorption of F127 onto the Ag NP surface at various concentrations (Figure 5a–e). SEM analysis of centrifuged deposits (Figure 6) revealed severe agglomeration and irregular morphology in the absence of F127. The addition of F127 significantly improved dispersion uniformity. However, at a high concentration of 12 wt.%, smaller particles were observed to adsorb onto larger ones, which can be attributed to depletion flocculation induced by free micelles or restricted particle mobility due to increased medium viscosity [39].

**Figure 7:**
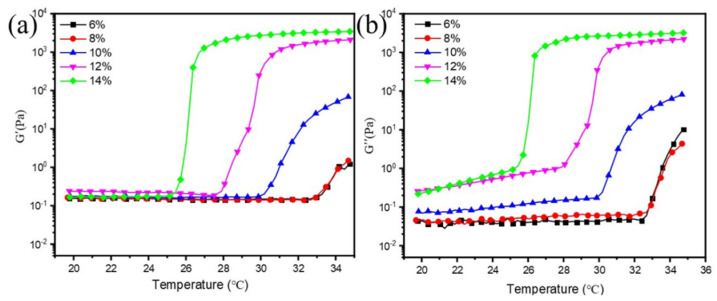
Effect of different amounts of F127 on rheological properties of Ag NP ink: (**a**) storage (G′) moduli; (**b**) loss (G″) moduli.

The influence of F127 content on the ink’s viscoelastic properties is shown in Figure 7. Increasing the F127 concentration from 6 to 14 wt.% systematically reduced the gel-sol transition temperature and increased both the storage (G′) and loss (G″) moduli. This indicates that F127 dominates the rheological behavior of the ink, forming a more structured fluid network at higher concentrations. The low values of G′ and G″ at room temperature, combined with the absence of a pronounced gel network, indicate that the ink behaves as a predominantly viscous fluid with minimal elasticity. This rheological profile is favorable for inkjet printing, as excessive elasticity can impede ligament breakup and lead to satellite droplet formation and jetting instability.

Based on the combined assessment of dispersion quality (TEM/SEM), resistance to flocculation, and rheological properties, a concentration of 10 wt.% F127 was identified as optimal. This concentration provided complete surface coverage and effective steric stabilization without inducing significant depletion flocculation or excessive viscosity, achieving an ideal balance between colloidal stability and printability.

### 3.4. Electrical Performance: Influence of Dispersant System and Sintering Temperature

**Figure 8:**
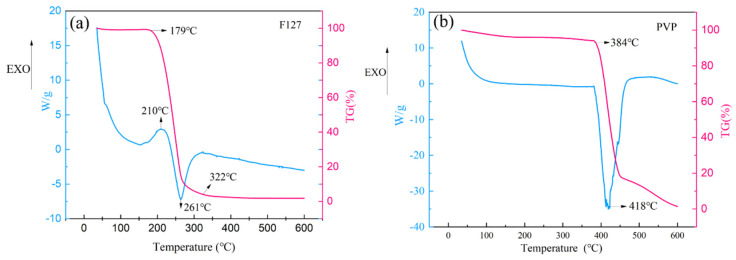
TG-DSC curves of F127 (**a**) and PVP (**b**), showing their thermal decomposition behaviors.

The electrical conductivity of the sintered film is largely governed by the extent of dispersant decomposition and removal. Thermogravimetric analysis indicated that neat F127 decomposes rapidly between 179 °C and 322 °C, whereas neat PVP begins decomposing at approximately 384 °C (Figure 8). This suggests a significant difference in their removal kinetics during the low-temperature sintering process.

**Figure 9:**
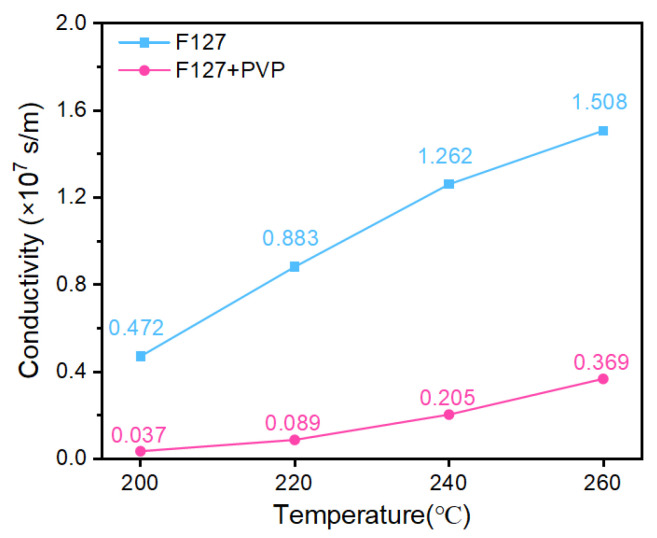
Comparison of the conductivity of Ag NP inks prepared with F127 alone and an F127/PVP composite dispersant as a function of sintering temperature.

Electrical measurements strongly reflected this anticipated difference (Figure 9). The ink formulated with 10 wt.% F127 as the sole dispersant (Table 1, Optimized ink) achieved a high conductivity of 1.506 × 10^7^ S/m after sintering at 260 °C. This vastly outperformed the separate F127/PVP composite system (Table 1, Composite ink) (0.369 × 10^7^ S/m) by approximately 4.1 times. The conductivity of the F127-based ink increased markedly as the sintering temperature rose from 200 to 260 °C, while the composite system showed a diminished improvement.

**Figure 10:**
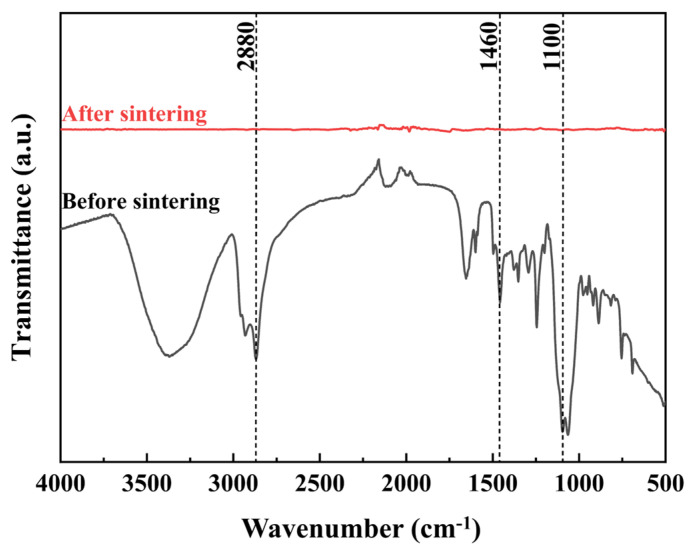
FTIR spectra of printed Ag NP ink with F127-only dispersant before and after sintering.

Direct evidence for the removal of the F127 dispersant was obtained via FTIR spectroscopy on the printed Ag NP ink with F127-only dispersant before and after sintering (Figure 10). The spectrum of the F127-based film after sintering at 260 °C showed the near-complete disappearance of characteristic organic peaks (e.g., C–H stretching ~2880 cm^−1^, C–H bending ~1460 cm^−1^, and C–O–C stretching ~1100 cm^−1^) vibrations [40], confirming the effective thermal decomposition and removal of F127. This successful removal facilitates the fusion of Ag NPs into a continuous network.

**Figure 11:**
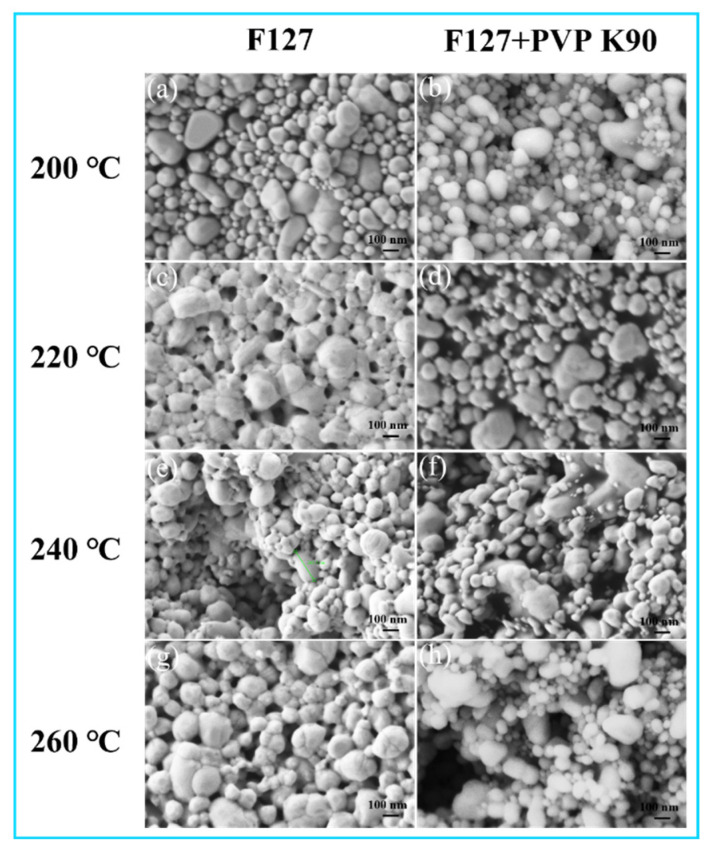
Comparison of SEM images of Ag NP inks prepared with a single F127 dispersant: (**a**) 200 °C, (**c**) 220 °C, (**e**) 240 °C, (**g**) 260 °C versus an F127/PVP composite dispersant system: (**b**) 200 °C, (**d**) 220 °C, (**f**) 240 °C, (**h**) 260 °C after sintering at various temperatures.

The inferior conductivity of the composite system is then reasonably attributed to the persistence of PVP residue, as inferred from its higher thermal stability shown by TGA. This interpretation is strongly supported by the corresponding microstructural evidence. SEM images (Figure 11) revealed that the F127-based ink formed a continuous and dense conductive network [41], whereas the film from the composite system exhibited a porous and defective morphology, consistent with the obstruction caused by undecomposed organic residue. Thus, the direct FTIR evidence confirms the clean removal of F127 at 260 °C, while the combined evidence from TGA, electrical performance, and SEM microstructure strongly suggests that residual PVP [28,29] impedes effective sintering in the composite system.

**Figure 12:**
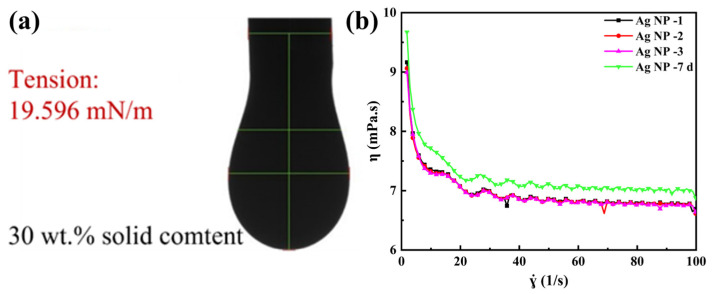
Surface tension (**a**) and viscosity (**b**) of the final optimized Ag NP ink.

Complementing the electrical and microstructural analyses, the rheological properties of the final optimized ink (Table 1, F127-only ink), critical for reliable jetting, were thoroughly characterized within a process-oriented framework. As shown in Figure 12b, the ink exhibited shear-thinning behavior over a broad shear rate range (1–100 s^−1^), which is beneficial for flow under the high shear within the printhead. However, within the intermediate shear rate range of 30–60 s^−1^—a range representative of the bulk flow in ink supply lines before droplet ejection [30]—the viscosity plateaued with minimal variation (<5%), indicating near-Newtonian behavior. This predictable flow response in the transfer stage is crucial for consistent droplet formation. The average viscosity measured at a representative shear rate of 40 s^−1^ was 6.83 mPa·s.

Furthermore, to assess practical handling and storage stability—a key consideration for printable functional inks [42]—the ink was aged under ambient conditions (25 °C) for 7 days. Subsequent measurement showed the viscosity at 40 s^−1^ remained within 3.54% of its initial value (Figure 12b). This excellent temporal stability, combined with the favorable shear-dependent behavior, confirms that the optimized ink possesses suitable and stable flow characteristics for robust inkjet printing. Together with a surface tension of 19.60 mN/m at 25.0 °C (Figure 12a), these properties confirm that the optimized ink possesses suitable and stable flow characteristics for robust inkjet printing.

### 3.5. Inkjet Printing Verification

#### 3.5.1. Quantitative Printability Assessment

A printer-independent assessment of the ink’s jetting capability was performed by calculating the Ohnesorge number (Oh), a dimensionless parameter that relates viscous, inertial, and capillary forces during droplet ejection: Oh = η/(γρα)^1/2^, where η is the viscosity (6.83 mPa.s, measured at a shear rate of 40 s^−1^ and 25.0 °C), γ is the static equilibrium surface tension (19.60 mN/m at 25.0 °C), ρ is the density (1120 kg/m^3^ at 25.0 °C), and α is the nozzle diameter (30 μm).

The calculated Oh value is 0.266, corresponding to Z = 1/Oh = 3.76. This Z number falls well within the widely accepted printable range of 1 < Z < 14 for stable droplet formation in piezoelectric inkjet printing [43,44], a criterion routinely applied to assess the jetting behavior of functional nanomaterial inks. This provides a quantitative, printer-independent foundation for the expected jetting stability.

#### 3.5.2. Printing Demonstration and Performance

**Figure 13:**
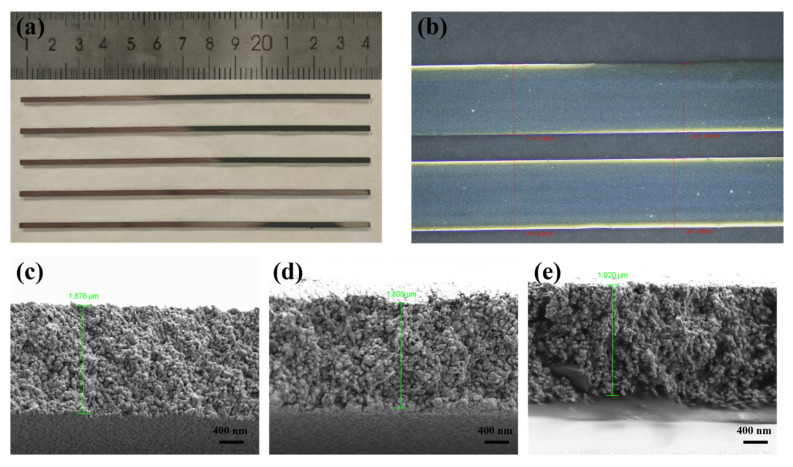
Printing and morphological characterization of the optimized Ag NP ink on a glass substrate: (**a**) Optical photograph of sintered conductive patterns; (**b**) Optical micrograph of a printed line; (**c**–**e**) Cross-sectional SEM images of a sintered Ag line.

The printability and functional performance of the optimized ink (formulated with Brofos Ag NPs, 10 wt.% F127, post-1500 r/min centrifugation, and HPH dispersion) were successfully demonstrated under the optimized conditions (29 μm drop spacing, single pass). Using the customized printer with a 30 μm nozzle, the ink was reliably jetted onto a glass substrate without clogging, producing well-defined and uniform lines and patterns (Figure 13a). After sintering at 260 °C for 1 h, these patterns exhibited a metallic luster and high conductivity, confirming the efficacy of the synergistic optimization strategy.

The geometrical and electrical characteristics [45] of the sintered lines printed under the optimized conditions (29 μm drop spacing, single pass) are summarized in Table 2. Optical microscopy confirmed a consistent line width of 1.20 ± 0.01 mm with no coffee-ring effect (Figure 13b), while cross-sectional SEM analysis revealed a uniform thickness of 1.87 ± 0.06 µm (Figure 13c–e). Within the studied parameter range, this morphological uniformity is attributed to the optimized solvent composition and the inclusion of glycerol, which promoted homogeneous drying by moderating Marangoni flows [7].

While the headline conductivity of 1.506 × 10^7^ S/m (Table 2) was achieved on glass and PI substrates after sintering at 260 °C, further evaluation of the ink’s compatibility with more heat-sensitive, common flexible substrates was conducted. To this end, the ink was also printed onto PI and PET. PI, which can withstand the standard 260 °C sintering process, yielded well-defined conductive lines. For the temperature-sensitive PET, a lower sintering temperature of 150 °C was applied, successfully demonstrating the ink’s printability and pattern formation on this substrate.

**Figure 14:**
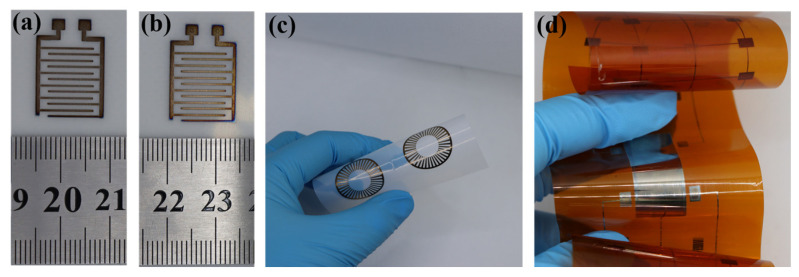
Demonstration of ink stability and compatibility with flexible substrates. (**a**,**b**) Optical images of a circuit printed on PET: (**a**) immediately after preparation and (**b**) after 7 days of ambient storage; (**c**,**d**) Optical photographs of sintered conductive lines under bending on (**c**) PET and (**d**) PI substrates.

The dense, continuous microstructure of the sintered film (Figure 11) and the near-complete removal of organics (Figure 10) are expected to contribute to good oxidation resistance and long-term electrical stability [46]. Furthermore, the ink itself exhibited excellent colloidal stability, maintaining its printability after 7 days of ambient storage (Figure 14a,b), which underscores its suitability for practical use under the studied conditions. The flexibility of the sintered patterns on both PET and PI substrates is demonstrated in Figure 14c and d, respectively.

The stable jetting performance of the optimized ink is substantiated by a convergence of evidence: (i) the theoretically calculated Z number (3.76) lies within the well-established stable printing range (1 < Z < 14); (ii) experimentally, clog-free continuous printing was sustained for over 2 h; and (iii) the resulting patterns, as shown in Figure 13a, are uniform and continuous. While direct high-speed imaging of droplets was not available, this combination of theoretical and experimental indicators provides strong support for reliable droplet formation under the optimized printing conditions.

## 4. Conclusions

This study successfully fabricated a highly conductive and stable inkjet-printable Ag NP ink by employing a synergistic strategy incorporating centrifugal classification, high-pressure homogenization, and optimized application of Pluronic F127. The findings underscore the crucial importance of a multi-parameter optimization approach and present a scalable and effective pathway for fabricating high-performance functional inks. The principal conclusions are as follows:

1. Centrifugal classification at 1500 r/min effectively selected Ag NPs (Brofos) with a narrow size distribution (>70% <50 nm), providing the foundational step for enhancing ink stability by reducing sedimentation driving forces.
2. High-pressure homogenization proved superior to acoustic resonance dispersion, generating stronger shear forces to achieve a more stable dispersion with lower and more stable viscosity, which is crucial for printability.
3. The key to achieving high conductivity lies in the selection of Pluronic F127 as the sole dispersant. It provided exceptional steric stability, and crucially, its relatively low decomposition temperature (~322 °C) allowed for near-complete removal during the 260 °C sintering process. This enabled extensive particle fusion into a dense network, yielding a high conductivity of (1.506 ± 0.08) × 10^7^ S/m (on glass/PI substrates) with 10 wt.% F127.
4. The synergistic integration of these optimized steps resulted in an ink with excellent overall performance under the specific conditions studied: appropriate rheology (viscosity = 6.83 mPa·s, surface tension = 19.60 mN/m), reliable inkjet printing stability under the optimized printing parameters, and high conductivity after low-temperature sintering, demonstrating significant potential for practical applications in printed electronics within this framework.

## Figures and Tables

**Table 1:** Composition of Ag NP ink variants studied in this work (in wt.%).

Component	Base Ink (with PVP)	Optimized Ink (F127 Only)	F127/PVP Composite Ink
Ag NPs (Brofos, modified)	30	30	30
PVP (K30)	4.5	0	2.25
Pluronic F127	0	10	5
TEGME	42.7	42.7	42.7
DI water	15	10	12.5
IPA	4.5	3	3.75
Ethanol	0.5	0.5	0.5
Glycerol	2.5	2.5	2.5
2-(2-ethoxyethoxy)ethyl acrylate	0.1	0.1	0.1
PVB	0.1	0.1	0.1
Defoamer K155	0.2	0.2	0.2
Total	100.0	100.0	100.0

**Table 2:** Geometrical and electrical characteristics of printed lines (optimized ink, sintered at 260 °C).

Parameter	Value (Mean ± SD)
Line width (mm)	1.20 ± 0.01
Thickness (μm)	1.87 ± 0.06
Sheet resistance (Ω/sq)	0.0355 ± 0.002
Conductivity (S/m)	(1.506 ± 0.08) × 10^7^
Coffee-ring effect	Not observed

## Data Availability

The original contributions presented in this study are included in the article. Further inquiries can be directed to the corresponding authors.
